# GRK2 contributes to glucose mediated calcium responses and insulin secretion in pancreatic islet cells

**DOI:** 10.1038/s41598-021-90253-z

**Published:** 2021-05-27

**Authors:** Jonathan Snyder, Atreju I Lackey, G. Schuyler Brown, Melisa Diaz, Tian Yuzhen, Priscila Y. Sato

**Affiliations:** Drexel University College of Medicine, Department of Pharmacology and Physiology, 245 N 15th Street, NCB 8119, Philadelphia, PA 19102 USA

**Keywords:** Heart failure, Diabetes, Cell signalling

## Abstract

Diabetes is a metabolic syndrome rooted in impaired insulin and/or glucagon secretory responses within the pancreatic islets of Langerhans (islets). Insulin secretion is primarily regulated by two key factors: glucose-mediated ATP production and G-protein coupled receptors (GPCRs) signaling. GPCR kinase 2 (GRK2), a key regulator of GPCRs, is reported to be downregulated in the pancreas of spontaneously obesogenic and diabetogenic mice (ob/ob). Moreover, recent studies have shown that GRK2 non-canonically localizes to the cardiac mitochondrion, where it can contribute to glucose metabolism. Thus, islet GRK2 may impact insulin secretion through either mechanism. Utilizing Min6 cells, a pancreatic ß-cell model, we knocked down GRK2 and measured glucose-mediated intracellular calcium responses and insulin secretion. Silencing of GRK2 attenuated calcium responses, which were rescued by pertussis toxin pre-treatment, suggesting a Gαi/o-dependent mechanism. Pancreatic deletion of GRK2 in mice resulted in glucose intolerance with diminished insulin secretion. These differences were due to diminished insulin release rather than decreased insulin content or gross differences in islet architecture. Furthermore, a high fat diet feeding regimen exacerbated the metabolic phenotype in this model. These results suggest a new role for pancreatic islet GRK2 in glucose-mediated insulin responses that is relevant to type 2 diabetes disease progression.

## Introduction

Peripheral tissue uptake of metabolic substrates, including glucose, is regulated by circulating insulin, glucagon, and other peptide hormones secreted by the pancreatic islet of Langerhans (islet). This process is essential for the utilization of glucose in peripheral tissues as islet dysregulation is a principal contributing factor in diabetes disease progression, resulting in mismatched insulin secretion and peripheral insulin sensitivity. To that end, insulin secretion is primarily regulated by increasing blood glucose and direct responses of ß-cells within pancreatic islets. Glucose primarily enters ß-cells though insulin-independent glucose transporter 2 (GLUT2) and is metabolized via glycolysis and oxidative phosphorylation to produce ATP. ATP inhibits ATP-sensitive potassium channels leading to the depolarization of ß-cells and activation of voltage-gated calcium channels promoting calcium-induced calcium release and insulin secretion^1,2^. This primary mechanism controlling insulin secretion is regulated by several pathways including G-protein coupled receptor (GPCR) activation, intermediary metabolite amplification, and secretory competency^3^.

GPCRs are a large and diverse family of receptors expressed throughout the body regulating a gamut of cellular processes. Specifically, in ß-cells, GPCRs modulate insulin secretion through multiple pathways impacting efflux of intracellular calcium stores and altering mitochondrial metabolism^4,5^. GPCR kinases (GRKs) are key regulators of activated GPCRs as GRKs phosphorylate the intracellular domain of GPCRs, leading to the recruitment of ß-arrestin and subsequent receptor internalization and desensitization. Although this is the canonical function of GRKs, other non-canonical functions have been implicated for GRKs in a vast array of cellular mechanisms^6^. In addition to complex signaling, the unique interactome for a GRK isoform appears to vary in a cell-specific manner. As such, in a highly specialized cell, with a unique physiological function and epigenetic profile, the interactome for islet GRK2 is unlike any other cell type and yet to be defined. Although human pancreatic islets express nearly 300 distinct GPCRs^7,8^ and various GPCRs modify pancreatic islet function^9–11^ little is known about GRK2’s islet-specific cellular function and its impact to cardiac metabolism and function.

GRK2 is widely expressed in the body with complex and unique tissue-specific responses to various pathologies. For instance, in the spontaneously obesogenic and diabetogenic mice, GRK2 is upregulated in the heart^12^ and downregulated in the pancreas^13^. The importance of GRK2 in physiology is exemplified by studies showing that global GRK2 deletion leads to embryonic lethality at day 15^14^, GRK2 inhibition impairs kidney function^15^, reduced GRK2 leads to a more severe phenotype of angioedema^16^, and cardiac-specific inhibition of GRK2 on a high fat diet regimen leads to an obesogenic phenotype^17^. Yet, in heart failure (HF) models, GRK2 inhibition either via global small molecule or cardiac specific peptide inhibitor restores functional adrenergic reserve and improves cardiac contractility while diminishing cardiac hypertrophy^18,19^. Moreover, global GRK2-peptide inhibition in a diabetic mouse model led to cardioprotection and improved glucose tolerance by improving insulin sensitivity and glucose utilization by the heart^20^. Of note, the latter study investigated metabolic responses to acute administration of GRK2 inhibitor peptides solely in diabetic mice, which may already express low levels of pancreatic GRK2. That is, the acute effect of GRK2 inhibitors short-term may improve diabetic metabolism by improving peripheral tissue glucose utilization; although the impact on non-diabetic animals was not investigated. Thus, understanding the role of GRK2 in metabolically-demanding and metabolite-sensing organs is imperative for the development of pharmacological strategies in patients with HF and diabetes as co-diagnosis. This study sheds novel insight into how GRK2 downregulation may be an adaptive or maladaptive response in insulin signaling. Utilizing a cell model for pancreatic islets and a novel mouse model of pancreatic-GRK2 donwregulation, we investigate the role of GRK2 in modulating glucose-mediated insulin responses. We propose that our data will provide new insights that could guide novel treatment strategies in a more tailored and personalized medicine approach for patients with HF and diabetes.

## Results

### Downregulation of GRK2 in Min6 cells leads to diminished glucose-mediated calcium responses and insulin secretion that are rescued by pertussis toxin

**Figure 1:**
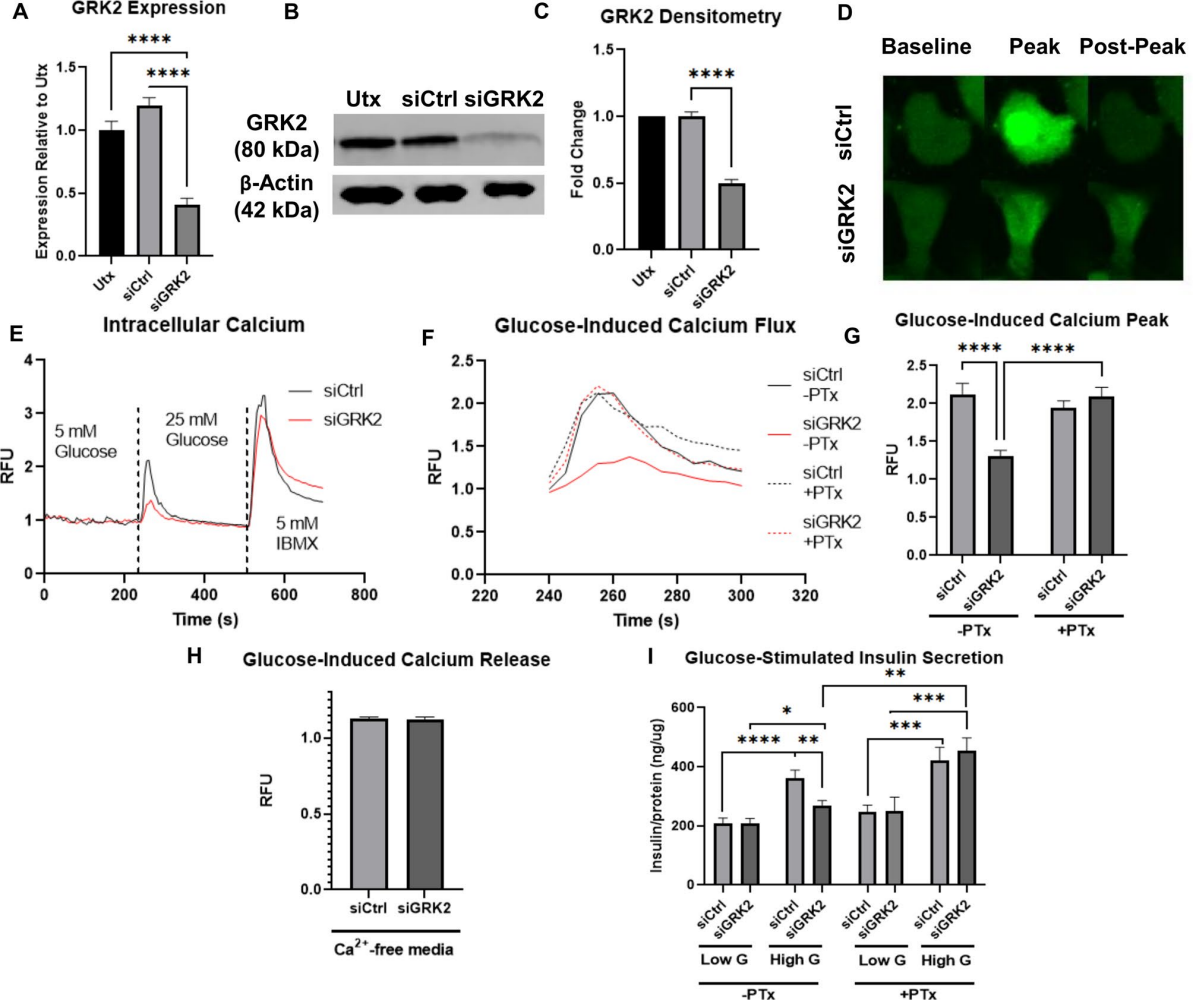
Reduction of GRK2 in Min6 cells diminishes glucose-mediated calcium signaling and insulin secretion. (**A**) GRK2 relative expression level in Min6 cells treated with silencing oligonucleotides (n = 10 samples/group in five independent experiments). (**B**) Representative Western blot analysis for GRK2 in Min6 cells untreated (Utx) or treated with scrambled control oligonucleotides (siCtrl) or GRK2-silencing oligonucleotides (siGRK2); full-length Western blot is shown in “Supplementary Material”. (**C**) Quantification of (**B**) (n = 16 samples/group in 16 independent cultures). Data shown as GRK2 over β-actin fold change (Fold Change) (**D**) Representative confocal images of glucose-induced calcium dependent fluorescence at frame 15 s post glucose injection. (**E**) Combined trace of experiments in (**D**) (n = 48–60 cells/group in four independent experiments): average calcium-dependent fluorescence normalized to baseline fluorescence. (**F**) Average glucose-induced calcium fluorescence signal in Min6 cells pre-treated with or without pertussis toxin (PTx, a Gα_i/o_ inhibitor). (**G**) Quantification of glucose-stimulated calcium signal peak in the presence or absence of PTx (n = 48–60 cells/group in 4–5 independent experiments). (**H**) Quantification of glucose-induced calcium peak in the absence of extracellular calcium (n = 70–72 cells/group in five independent experiments). (**I**) Glucose-stimulated insulin secretion normalized to total protein content from cells treated with low glucose (3 mM) or high glucose (16.7 mM) with or without PTx pre-treatment (n = 12/group in four independent experiments). Data shown as mean ± SEM. *p ≤ 0.05; **p ≤ 0.01; ***p ≤ 0.001; ****p ≤ 0.0001.

GRK2 expression in Min6 cells was decreased by more than 50% at message level (Fig. 1A) and corresponding protein level (Fig. 1B,C and Supplementary Fig. 1A,B) when compared to control (siCtrl) cells. GRK2 expression level was comparable in siCtrl and untreated (Utx) cells (Fig. 1A–C) with no GRK isoform compensation in GRK2 knockdown (siGRK2) cells (Supplementary Fig. 1C). To elicit acute calcium responses, cells were exposed to glucose where intracellular calcium was measured using a temperature-controlled confocal microscope. Calcium responses were diminished in siGRK2 cells when compared to siCtrl cells in response to glucose elevation (Fig. 1D,E). Moreover, cytosolic calcium increases were comparable in response to IBMX in both conditions (Fig. 1E and Supplementary Fig. 1D) suggesting similar calcium stores in siGRK2 cells when compared to control. Glucose-stimulated calcium signal was rescued in siGRK2 cells by pretreatment with Gαi/o inhibitor pertussis toxin (PTx) suggesting a Gαi/o-involvement for GRK2 function in the islet (Fig. 1F,G). Silencing of GRK2 did not impact intracellular-restricted calcium release (Fig. 1H and Supplementary Fig. 1E). Glucose-stimulated insulin secretion was diminished in siGRK2 cells when compared to control cells. siGRK2 cells in high glucose had diminished insulin secretion when compared to siCtrl cells that was rescued with PTx pre-treatment (Fig. 1I and Supplementary Fig. 1F). Glucose-induced cAMP levels in siGRK2 cells were comparable to control cells when in the presence of IBMX pre-treatment (Supplementary Fig. 1G). These data suggest that islet GRK2 regulates glucose-mediated calcium signaling via Gαi/o, that is relevant to calcium influx.

### Decreased levels of GRK2 in Min6 cells attenuate mitochondrial coupling efficiency, increase proton leak, and decrease ATP levels

**Figure 2:**
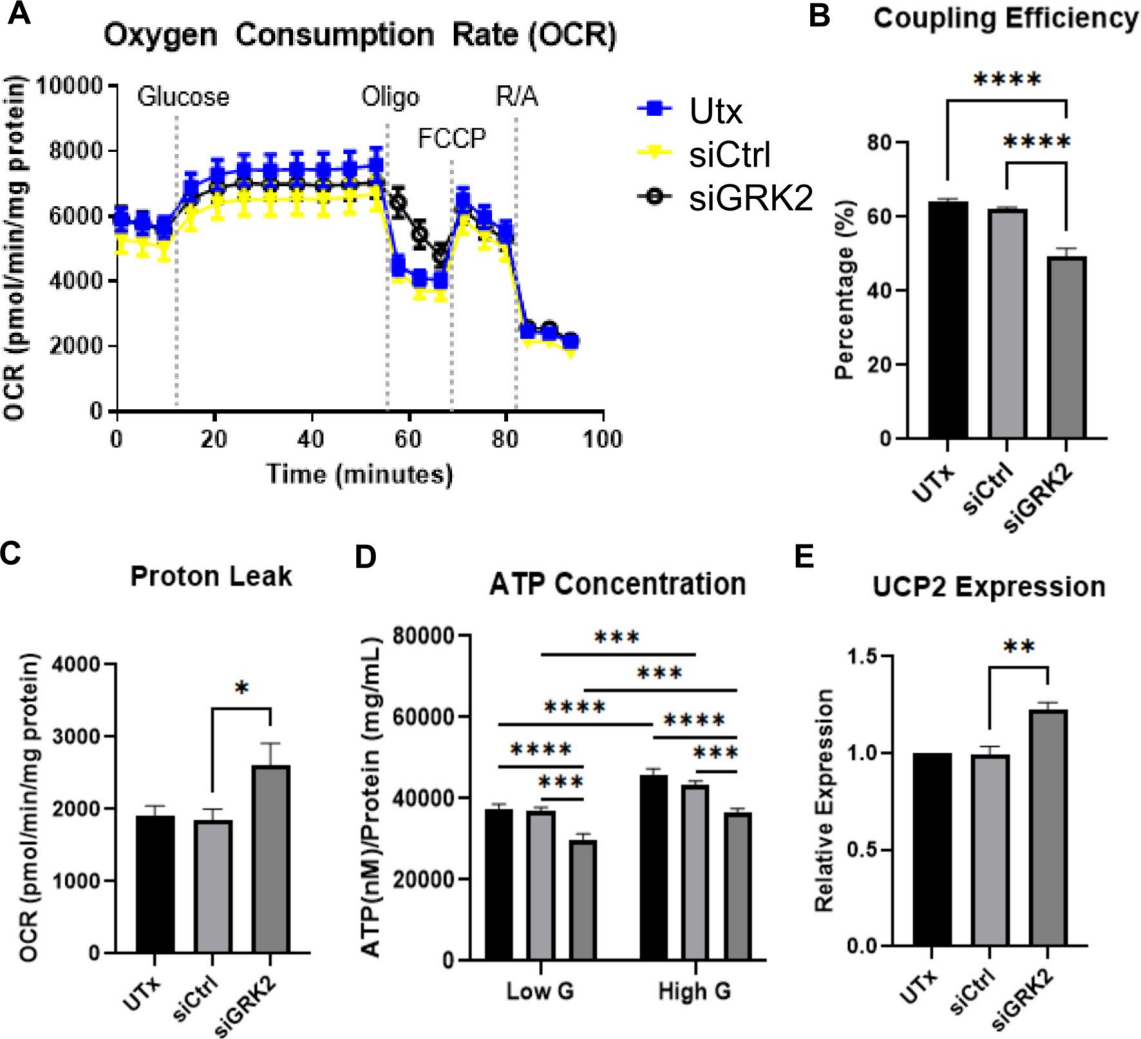
Reduced GRK2 in Min6 cells alters mitochondrial function. (**A**) Overall oxygen consumption rates (OCR) in cells stimulated with glucose followed by mitochondrial stress test (n = 24 samples/group in three independent experiments; mean ± SEM). (**B**) Coupling efficiency of experiments in (**A**). This is the percentage ratio of ATP-synthase dependent respiratory rate over basal respiratory rate (same n values as **A**). (**C**) Quantification of proton leak (same n values as panel A). (**D**) Luciferin-based ATP measurements in Min6 cells normalized to total protein concentration (n = 8 recordings/group in eight independent cultures). (**E**) UCP2 expression level in Min6 cells relative to 18 s housekeeping gene (n = 8 preparations/group in eight independent cultures). Data shown as mean + /-SEM. *p ≤ 0.05; **p ≤ 0.01; ***p ≤ 0.001; ****p ≤ 0.0001.

To investigate the impact of GRK2 knockdown on glucose-mediated islet mitochondrial responses, we performed Seahorse oxygen consumption rate (OCR) analysis using the first port to deliver high levels of glucose (Fig. 2A). OCR responses due to glucose were comparable among the groups yet coupling efficiency in siGRK2 cells was reduced (Fig. 2B) with increased proton leak (Fig. 2C). Baseline, maximal respiration, and non-mitochondrial OCRs were not statistically different (Fig. 2A). Likewise, luciferin-based ATP determination in siGRK2 cells maintained in low glucose (3 mM; Low G) or exposed to high glucose (16.7 mM; High G) showed decreased ATP levels when compared to siCtrl cells (Fig. 2D). Moreover, uncoupling protein 2 (UCP2) levels were increased in siGRK2 cells when compared to siCtrl (Fig. 2E). This was without any alterations in UCP3, premature, or mature insulin (Supplementary Fig. 2A), reactive oxygen species (Supplementary Fig. 2B), or lipid peroxidation (Supplementary Fig. 2C). No statistical difference was observed in electron transport chain complex protein expression (Supplementary Fig. 2D–F) nor ATP subunit c protein expression (Supplementary Fig. 2G–I). Thus, loss of islet GRK2 attenuates mitochondrial efficiency and ATP levels.

### Loss of GRK2 in murine pancreas promotes glucose intolerance, diminishes cardiac function, and impairs insulin release

**Figure 3:**
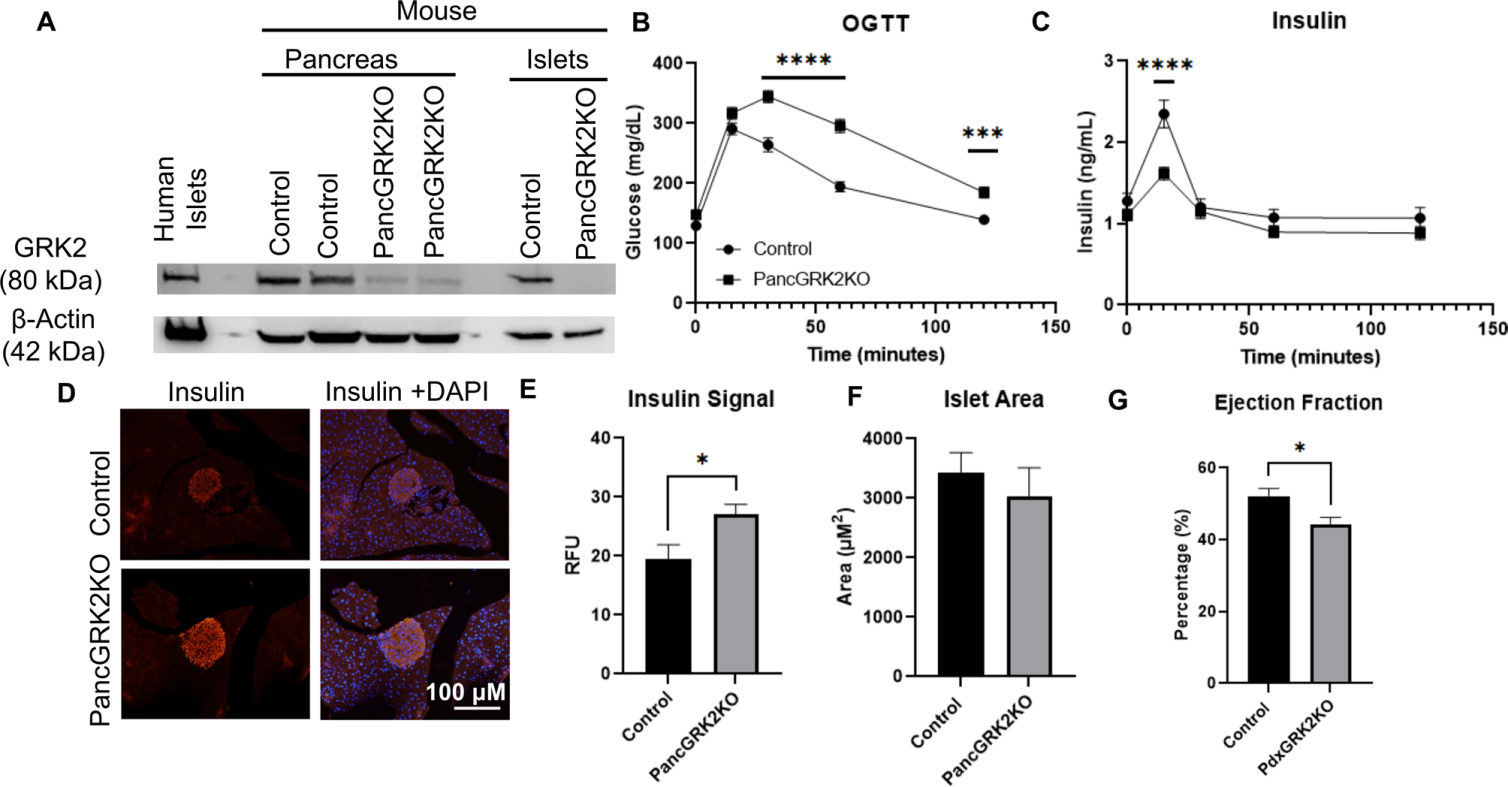
Pancreatic GRK2 knockdown leads to glucose intolerance via impaired insulin secretion leading to attenuated cardiac function. (**A**) Representative GRK2 Western blot analysis from pancreas and isolated islets from pancGRK2KO mice; full-length Western blot is shown in “Supplementary Material”. (**B**) Overall tracing of blood glucose levels during oral glucose tolerance test (OGTT) in control (circles) and pancGRK2KO mice (squares) (n = 15–22 animals/group). (**C**) Serum insulin measurements in animals corresponding to OGTT time points in (**B**) (same n values as in **B**). (**D**) Representative immunofluorescence images for insulin and insulin with nuclear staining (DAPI). (**E**) Insulin immunofluorescence signal quantification (n = 29–32 images/group in 4–5 animals/group). (**F**) Islet area quantification in µm^2^ (n = 51–69 islets/group in 4–5 animals/group). (**G**) In vivo ejection fraction quantification (n = 8 hearts/group). Data shown as mean ± SEM. *p ≤ 0.05; ***p ≤ 0.001; ****p ≤ 0.0001.

GRK2 is expressed in human islets and pancreatic (pdx-driven)-knockout of GRK2 (pancGRK2KO) led to more than 95% decrease in GRK2 protein levels in isolated murine islets (Fig. 3A, Supplementary Figs. 3 and 4A). Importantly, no statistical difference in glucose tolerance was observed in both controls for the line (Supplementary Fig. 4B) thus both genotypes (cre+ wt/wt or cre− flox/flox) were grouped and used as controls for all experiments. PancGRK2KO animals were glucose intolerant when compared to control animals (Fig. 3B) with decreased serum insulin (Fig. 3C) at 15 min post-glucose gavage. Insulin tolerance was comparable among both groups (Supplementary Fig. 4C). Intracellular insulin levels were statistically increased in pancGRK2KO islets when compared to control animals (Fig. 3D,E) with no statistical alterations in glucagon levels (Supplementary Fig. 4D–F) nor islet area (Fig. 3F). Moreover, no statistical difference was observed in message level for premature insulin, mature insulin, or α2AR (Supplementary Fig. 4G). Diminished cardiac function in pancGRK2KO mice was observed when compared to control mice (Fig. 3G) that was not linked to gross signs of cardiac hypertrophy (Supplementary Fig. 4H). Overall, these data suggest that pancreatic GRK2 participates in glucose-mediated insulin release that is linked to metabolic signaling to the heart.

### High fat diet challenge exacerbates pancGRK2KO glucose intolerant phenotype with enhanced weight gain and increased fat depots

**Figure 4:**
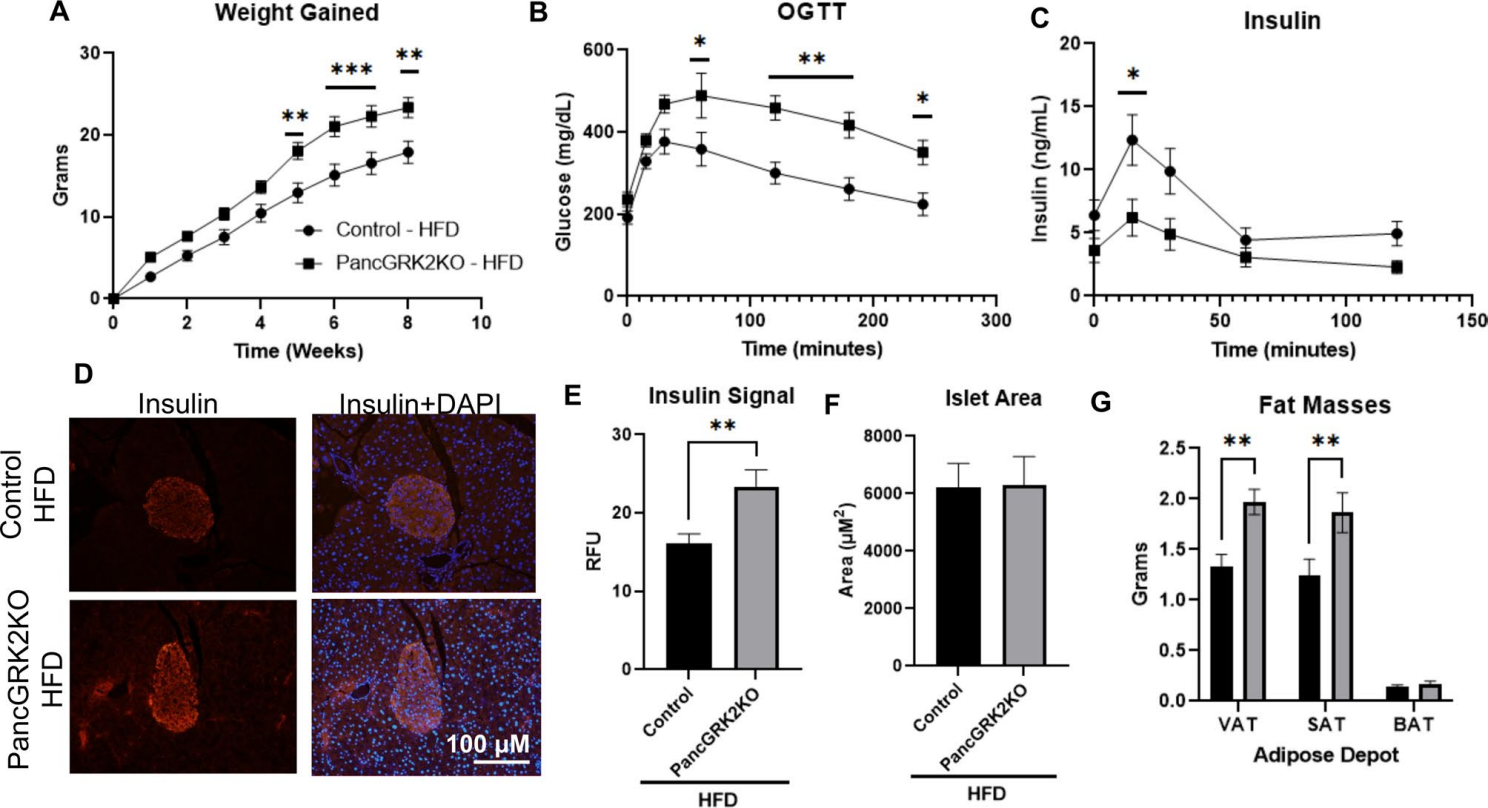
Exacerbation of metabolic dysfunction in pancGRK2KO under HFD regimen. (**A**) Weight gained over 8 weeks in mice under HFD (n = 10–14 animals/group Circles = control + HFD, Squares = PancGRK2KO + HFD). (**B**) Overall tracing of blood glucose levels during oral glucose tolerance test (OGTT) in control and pancGRK2KO mice post 8-weeks of HFD treatment (n = 10–14 animals/group) (**C**) Serum insulin measurements in animals corresponding to OGTT time points in (**B**) (same n values as in **B**). (**D**) Representative immunofluorescence images for insulin and insulin with nuclear staining (DAPI) in animals subjected to HFD. (**E**) Insulin immunofluorescence signal quantification (n = 24–34 islets/group in 4–5 animals/group (**F**) Islet area quantification in µm^2^ (n = 56–65 islets/group in 4–5 animals/group). (**G**) Masses of different adipose depots at the end of the 8-week period (n = 10–14 animals/group): *VAT* visceral epididymal adipose tissue, *SAT* subcutaneous inguinal adipose tissue, and *BAT* brown subscapular adipose tissue). Data shown as mean ± SEM. *p ≤ 0.05; **p ≤ 0.01; ***p ≤ 0.001.

Mice were metabolically challenged with a high fat diet (HFD) regimen where loss of pancreatic GRK2 led to statistically significant increases in weight gain (Fig. 4A) with slightly lower weekly food intake (Supplementary Fig. 5A). PancGRK2KO mice under HFD possessed an exacerbated glucose intolerant phenotype (Fig. 4B) and diminished insulin release (Fig. 4C) when compared to control animals. Insulin tolerance was not altered in pancGRK2KO under HFD (Supplementary Fig. 5B). In agreement with regular chow data, islet insulin levels were statistically increased in pancGRK2KO HFD islets (Fig. 4D,E) with no statistically significant alterations in glucagon levels (Supplementary Fig. 5C–E). HFD led to overall islet hyperplasia when compared to normal chow diet, but no statistical difference was observed in islet area due to genotype differences within the HFD group (Fig. 4F). Visceral adipose tissue (VAT) from the epidydimal fat depot and subcutaneous adipose tissue (SAT) from the inguinal fat depot were increased in pancGRK2KO HFD when compared with controls HFD animals (Fig. 4G). Subscapular brown adipose tissue (BAT) was not statistically different between groups. Overall, our data suggest that HFD promotes metabolic dysfunction in pancGRK2KO mice with increased VAT and SAT fat depots.

## Discussion and conclusion

In the present study we show a distinct functional role for pancreatic GRK2 where this kinase participates in regulating islet function. Reducing GRK2 in pancreatic cells (Fig. 1A–C) led to decreased glucose-mediated intracellular calcium signaling (Fig. 1D,E). This reduction is specific to glucose stimulation as treatment with IBMX, a non-selective phosphodiesterase inhibitor, leads to equal release of calcium stores indicating that calcium is equally available to be released in the siGRK2 cells during glucose stimulation (Fig. 1E and Supplementary Fig. 1D). Notably, cAMP measurements in the presence of IBMX is comparable between groups (Supplementary Fig. 1G), which is consistent with the reversed effect of IBMX on cytosolic calcium in siGRK2 cells (Fig. 1E). Interestingly, the reduction in glucose-mediated calcium signal is rescued by PTx, a Gαi/o inhibitor, suggesting that reduction of GRK2 promotes Gαi/o-inhibitory pathways (Fig. 1F,G). This is also substantiated by glucose-stimulated insulin secretion measurements (Fig. 1I). Thus, pancreatic GRK2 may regulate glucose-mediated calcium signaling by fine-tuning Gαi/o signaling. Gαi/o participates in norepinephrine (NE)-induced enhancement of open probability of K_ATP_ channels leading to diminished insulin secretion. This NE effect was abolished in cells preincubated with PTx, indicating participation of Gαi/o proteins^21^. Moreover, islet studies have shown a Gαi/o-dependent mechanism for the activation of a sulphonylurea-sensitive low conductance K channel that is distinct from K_ATP_ channels^22^. Activity of this low conductance K channel was confirmed in SUR1-knockout animals which do not possess active K_ATP_ channels^23^. Canonically, GRK2 is involved in GPCR desensitization and a vast number of islet GPCRs have been shown to decrease insulin signaling by promoting Gαi/o, including alpha 2 adrenergic receptor (α2AR^9^), FFAR2^10^, FFAR3^10^, mGluR3^24^, and somatostatin receptor^11^. Acutely, Gαi/o-coupled signaling impacts insulin secretion by altering glucose metabolism and electrical excitability; chronically, Gαi/o-coupled signaling can modify β-cell proliferation and gene expression patterns^25^. GRK2 is expressed in human and murine islets (Fig. 3A) and it is abundantly expressed in the islet relative to other GRK isoforms (Supplementary Fig. 1C). Therefore, it is plausible that loss of GRK2 may increase plasma membrane expression level of any of the above-mentioned Gαi/o-coupled receptors and/or prolong GPCR agonism through altered desensitization kinetics. This is similar to the detrimental outcome observed when GRK2 is reduced in endothelial cells, augmenting BK channel function and leading to vascular permeability and angioedema^16^. Further experiments will determine the upstream involvement of specific islet GPCRs in eliciting GRK2-mediated Gαi/o-signaling in insulin secretion and/or the newly described g-protein intracellular signaling activation and involvement in exocytosis^26^.

Furthermore, we have previously shown that GRK2 can localize to the cardiac mitochondrion where it regulates glucose utilization^27^. Due to the essential role of glucose metabolism in insulin secretion, we investigated mitochondrial function in islet cells with reduced GRK2. We emulated acute increases in glucose levels in these cells by diminishing media glucose followed by a sudden rise in extracellular glucose. Acute glucose-mediated mitochondrial respiration was measured over an hour followed by a mitochondrial stress test. Glucose-stimulated OCR was comparable among the groups (Fig. 2A). However, coupling efficiency was diminished in siGRK2 cells (Fig. 2A–C), with an attenuated response to oligomycin treatment, an ATP-synthase inhibitor. Likewise, luciferin-based ATP measurements revealed a reduction in ATP levels in siGRK2 cells post-glucose stimulation (Fig. 2D). This agrees with a previous study showing that reduction of GRK2 diminishes ATP levels in aorta-derived cells^28^. Our observations were in the absence of any major alteration in ROS production or lipid peroxidation (Supplementary Fig. 2B,C), suggesting that oxygen was not being diverted to other cellular regions. As proton leak was increased in our measurements (Fig. 2C), we determined levels of uncoupling proteins (UCP) which are known mediators of proton gradient dissipation. UCP2 and UCP3 are expressed in the pancreas, whereas UCP2 protein expression is restricted to few tissues including the endocrine system and is the predominant isoform in islets^29^. Reduced levels of islet GRK2 increased UCP2 expression (Fig. 2E) with no alterations in UCP3 (Supplementary Fig. 2A). This is consistent with reports showing UCP2 upregulation in human islets in metabolically adverse contexts such as hyperglycemia and/or lipotoxicity^29^ and reports showing that UCP2 knockout led to augmented glucose-stimulated insulin secretion^30^. No statistical difference was observed in the expression of electron transport chain complexes (Supplementary Fig. 2D–F) nor ATP synthase subunit c (Supplementary Fig. 2G–I). ATP synthase subunit c has been reported to be the pore forming unit of the mitochondrial permeability transition pore (mPTP)^31^ and we have shown that cardiac overexpression of GRK2 leads to an increased propensity for premature opening of the mPTP pore^32^. Although no observable alteration in ATP synthase subunit c expression is detected in siGRK2 cells, future studies will delineate the functional outcome of islet GRK2 in mPTP opening and calcium buffering mechanisms. Importantly, the observed decrease in ATP levels in siGRK2 cells (Fig. 2) in conjunction with the intracellular calcium data (Fig. 1) suggest a role for GRK2 in regulating calcium influx that is mediated by Gαi/o and ATP levels.

To substantiate our findings in Min6 cells, we constructed a novel pancGRK2KO mouse model. Important to reiterate is the fact that global GRK2 knockout mice are embryonically lethal at day 15^14^. No changes in glucose tolerance was observed in control animals whether or not they expressed cre-recombinase (Supplementary Fig. 4B). With almost 95% loss of GRK2 (Fig. 3A, Supplementary Figs. 3, and 4A), pancGRK2KO mice are viable, yet exhibit glucose intolerance with decreased insulin release following glucose administration (Fig. 3B,C). This is consistent with results of a microfluidic islet GPCR stimulation model that showed that enhanced Gαi or Gαq stimulation diminished glucose-stimulated but not basal insulin secretion^5^. The sensibility of diminished stimulated but not basal insulin secretion is further supported by a recent study that showed basal insulin secretion is not primarily dependent on ATP production^33^. Moreover, pancGRK2KO islets possessed increased intracellular insulin levels with no significant changes in islet area (Fig. 3D–F) nor glucagon levels (Supplementary Fig. 4D,E), suggesting a potential role for GRK2 specifically in β-cells of the islet. Importantly, no significant alterations were observed in pre-mature and mature insulin message (Supplementary Fig. 4G), suggesting no major alteration for insulin at the gene expression/processing level but rather ineffective insulin secretion. PancGRK2KO cardiac function was decreased with no gross indication of myocardial hypertrophy. This suggests that glucose intolerance may metabolically impact the heart even when insulin resistance is not a combinatory contributing factor. A fact also exemplified in type 1 diabetic patients who are also uniquely at risk for certain cardiac conditions such as diabetic cardiovascular autonomic neuropathy^34^.

To metabolically challenge these animals, we subjected the pancGRK2KO to HFD treatment where an increased weight gain was observed in pancGRK2KO animals compared to controls (Fig. 4A). This was associated with worsened glucose tolerance and decreased insulin release during OGTT (Fig. 4A–C). In agreement with normal chow findings, pancGRK2KO HFD had increased islet insulin levels (Fig. 4D,E) with no alteration in islet area (Fig. 4F) nor glucagon levels (Supplementary Fig. 5C–E) when compared to control HFD animals. HFD aggravated metabolic dysfunction in pancGRK2KO mice, leading to an augmented glucose intolerant phenotype that suggested an impairment in insulin release rather than production. Moreover, weight gain in pancGRK2KO mice did not appear to be evenly distributed but associated with an augmented increase in fat pad in VAT and SAT but not BAT (Fig. 4G). The functional consequences of such differences in adipose tissue accumulation are of interest for future studies.

**Figure 5:**
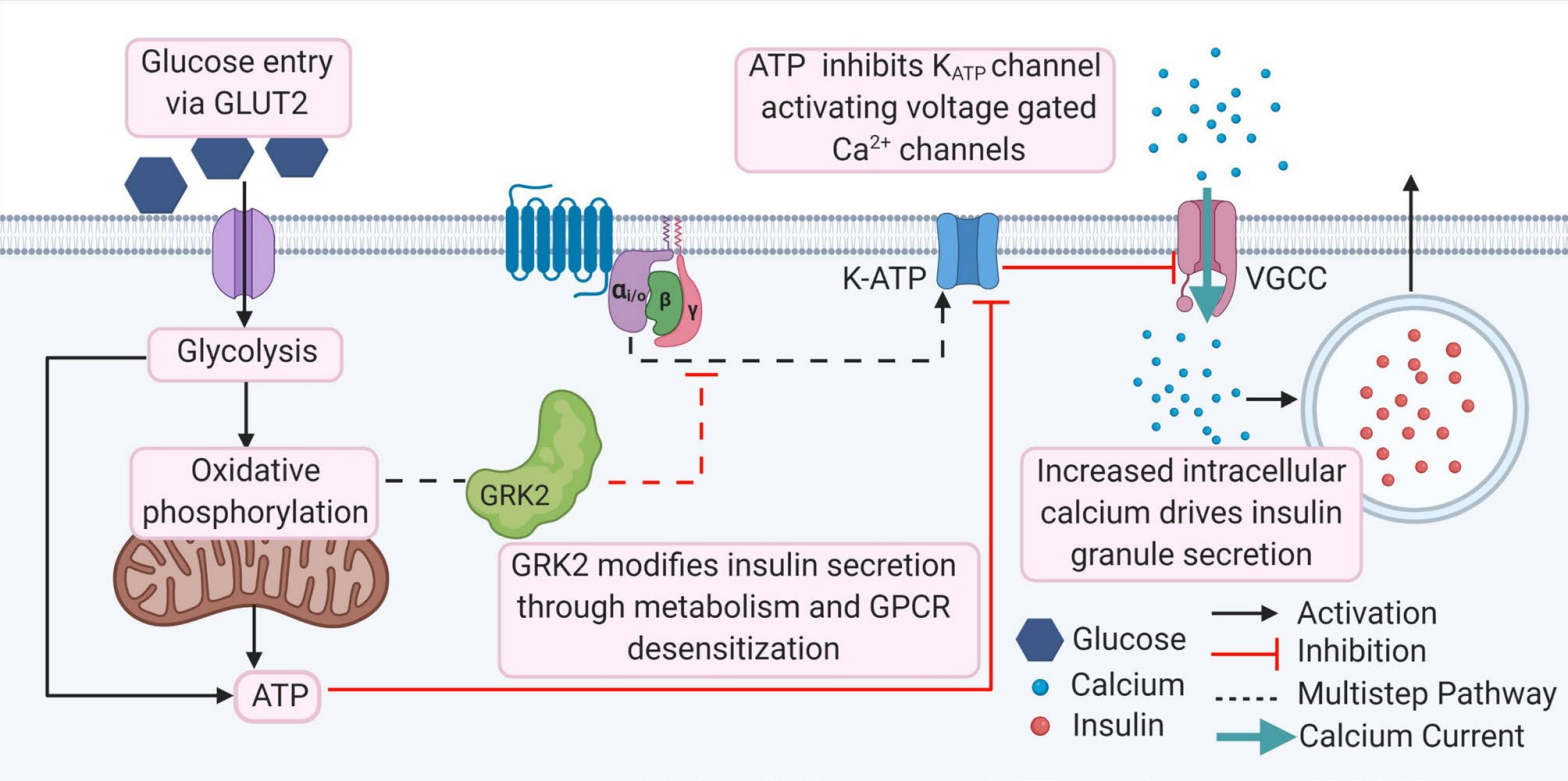
Schematic representation of mechanistic roles that GRK2 may play in islet cells. Created with biorender.com (https://www.biorender.com).

Overall, this study investigated how pancreatic GRK2 participates in islet function, in particular glucose-mediated responses leading to insulin release. Our data suggest that pancreatic GRK2 alters ATP levels which impact calcium influx in addition to Gαi/o-mediated signaling pathways that regulate insulin secretion from the islet. Schematic representation of our proposed model is shown in Fig. 5 wherein GRK2 under normal circumstances keeps Gαi/o-signaling in check. In its absence Gαi/o-signaling is less restrained increasing its inhibitory tone and diminishing calcium-induced insulin secretion. Both calcium flux and GRK2 itself are known regulators of mitochondrial function that may lead to respiratory inefficiency. These pathways converge on canonical calcium-mediated insulin secretion which we show to be diminished in this model.

Important to note, pancreatic GRK2 downregulation in this mouse model is at the embryonic-stage level and developmental alterations may play a factor in our observed results. Likewise, our findings in the cell line do not exclude the possibility of a role for GRK2 that is dependent on cell proliferation or accumulation of paracrine signaling molecules in the media. Yet, it is imperative to better understand how fine-tuning GRK2 may differentially impact specific tissues as GRK2 signaling appears to distinctively impact the function of islets, a highly specialized group of cells with a unique epigenetic profile. As preventing HF-induced GRK2 cardiac upregulation protects the heart from pathological dysfunction^35^, our study compels for systemic analysis when considering GRK2 inhibition as a mode of HF pharmacological treatment, particularly in pre-diabetic and diabetic patients. Studies investigating the loss of pancreatic GRK2 in an inducible cell-specific manner will be key to delineate the importance of this kinase in developmental versus adult stage insulin signaling. Additionally, unraveling islet cell-type specific GRK2 interactome could shed light onto novel and unexplored pharmacological GPCR candidates that could be exploited to benefit HF patients with diabetes.

## Materials and methods

### Min6 cell culture

Min6 cells were cultured in Addexbio optimized DMEM supplemented with 15% FBS and 50 µM β-mercaptoethanol and utilized up to passage 25. Min6 cells were maintained in a humidified incubator at 37 °C and 5% CO_2_. Cells were transfected as reported^36^ with Dharmafect 1 reagent following the manufacturer’s protocol (Horizon Discovery) and siRNA oligonucleotide complementary to GRK2 (siGRK2) or a scrambled oligonucleotide (siCtrl; Dharmacon technologies). For all experiments GRK2 knockdown was confirmed by either via RT-qPCR or Western blotting as detailed below.

### Generation of pancreatic GRK2-KO mice

All animal experimental procedures were approved by the Institutional Animal Care and Use Committee of the Drexel University College of Medicine, and all methods were performed in accordance with the relevant guidelines and regulations. The study was carried out in compliance with the Animal Research: Reporting of In Vivo Experiments (ARRIVE) guidelines. Mice were housed under pathogen-free conditions with environmental enrichment, with up to 5 mice per cage. GRK2 flox mice^37^, with GRK2 exons flanked by LoxP sites, were crossed with mice expressing cre-recombinase under the Pdx1-cre promoter (the Jackson Laboratory, Bar Harbor, ME, USA). Genotype was determined for the presence of flox and cre-recombinase using ear notches as previously performed^37^. For our studies male mice were used at 9–13 weeks of age unless otherwise stated.

### Primary pancreatic islet isolation

Islet isolation was performed as previously described^38^. Briefly, the common bile duct was ligated and the ampula of Vater perfused with HBSS containing collagenase (Sigma). Pancreas was excised and further digested with intermittent shaking at 37 °C. Digestion was stopped using HBSS containing 1 mM calcium and filtered using a 70 µm cell strainer. Islets were then hand-picked using a Leica S9i Stereoscope and cultured for 1 day in RPMI 1640 with 10% FBS, 1% penicillin/streptomycin, and 2 mM glutamine to allow for recovery from digestion prior to harvesting protein or RNA.

### Preparation of lysates and Western blot analysis

Western blots were performed as previously described^27^. Briefly, cells were harvested and lysed in RIPA buffer. Protein determination was performed using BCA (Pierce) and 30 µg of protein loaded onto tris–glycine or tricine gels. Primary antibodies (Supplementary Table 1) were incubated overnight at 4 °C, washed three times in PBS with Tween-20, incubated with secondary antibody for 1 h at room temperature, and washed three times. Imaging was performed using a LI-COR Odyssey Fc and analysis was performed off-line using Image Studio.

### RNA isolation and quantitative real-time PCR

RNA was extracted from Min6 cells or primary islets via Purelink™ RNA mini-kit (Ambion). RNA to cDNA conversion was performed using High-Capacity cDNA conversion kit (ThermoFisher). qPCR reactions were performed with TaqMan reagents for GRK isotypes while other qPCR reactions were performed using SYBR Green chemistry using 25 ng cDNA/reaction. All reactions were performed in triplicate using a QuantStudio 7 and ΔΔCt values were calculated using 18 s as the housekeeping gene. Primer sequences are displayed in Supplementary Tables 2 and 3.

### Cellular calcium imaging

Calcium imaging was performed based on published methodology^39^ and Fluo-8 calcium sensitive dye in Min6 cells as per manufacturer’s protocol (Abcam: ab112129). Briefly, transfected cells plated on a 35 mm Mattek glass bottom dishes coated with collagen were starved for 2 h in substrate limited media (DMEM + 1 mM sodium pyruvate + 2 mM glutamine), then loaded with Fluo-8 for 30 min in Tyrode solution (NaCl: 140 mM; KCl: 5.4 mM; CaCl_2_:1.8 mM;MgCl_2_: 1 mM; NaH2PO4: 0.33 mM; glucose: 5 mM; HEPES: 5 mM; pH = 7.4). Non-incorporated dye was washed off with Tyrode solution for total calcium flux experiments or with Tyrode solution without calcium and the addition of 100 µM EGTA for calcium release experiments. Cells were imaged every 5 s on a temperature-controlled Olympus FV1000 confocal microscope using a 60× objective. Glucose and IBMX were introduced by bath application. Quantification was performed using ImageJ.

### Glucose-stimulated insulin secretion

Min6 cells were equilibrated to 3 mM glucose in HBSS deprived of other substrates for 2 h. The supernatant was then removed and replaced with either fresh 3 mM glucose in HBSS (low glucose) or 16.7 mM glucose in HBSS (high glucose). The cells were incubated in these conditions for 30 min. Then, the supernatant was removed and immediately frozen in liquid nitrogen. Cells were lysed with RIPA solution and sonicated. The lysate was used for protein quantification. The Cisbio homogenous time-resolved fluorescence ultra-sensitive insulin assay kit was used according to manufacturer's protocol to measure insulin in supernatants with an EnSpire Envision plate reader. Insulin secretion was normalized to cellular protein content.

### cAMP measurements

Min6 cells were incubated in substrate-limited media for 2 h at 37 °C, followed by 30 min incubation in HBSS containing calcium in the presence of IBMX (500 μM). Cells were exposed to 16.7 mM glucose for 30 min, washed once with PBS, and prepared for cAMP measurements per manufacturer’s protocol (K019-Arbor Assays). Forskolin (100 μM) was incubated for 15 min in untreated cells as positive control. Samples were prepared and analyzed using the high sensitivity protocol and measured at 450 nm using a plate reader. Data analysis was performed offline using myassays software provided by Arbor Assays.

### Analysis of mitochondrial function via seahorse respirometry

Seahorse respirometry was performed with Min6 cells using an Agilent seahorse XF96e analyzer. Cellular and drug titration optimization experiments were performed to ensure maximal responses. Cells were incubated for 2 h prior to the experiment in substrate limited media, followed by Seahorse base media containing 3 mM glucose one hour prior to the initiation of recordings. Cellular stimulation was induced by glucose (16.7 mM), Oligomycin (1 µM), FCCP (1 µM), and rotenone/antimycin A (R/A; 1 µM). Protein determination was assessed at the end of the experiment (ThermoFisher) to normalize recordings. Analysis was performed off-line using Wave 2.6.1 software. Coupling efficiency was calculated using pre-set Wave calculations in normalized recordings, that is coupling efficiency was the ratio of ATP-driven respiratory rates over baseline respiratory rates (rate 3) × 100. Proton leak was calculated based on the difference between non-mitochondrial OCR (Post-R/A) and non ATP-synthase dependent OCR (Post-Oligo) respiration rates. No wells were excluded from the data presented.

### Reactive oxygen species (ROS)

Quantification of intracellular ROS in Min6 cells was performed using a dichlorofluorescein dye fluorescence-based detection of ROS, following manufacturer’s protocol (Abcam; ab113851). Cells were exposed to substrate limited media, then given 3 mM or 16.7 mM glucose for 45 min. Fluorescence was determined using a Tecan Spark Multi-mode plate reader. Reactions were performed in quadruplicate technical repeats.

### Lipid peroxidation assay

Peroxidated lipids were quantified by the Lipid Peroxidation Assay kit following manufacturer’s protocol (Abcam: ab118970). Briefly, Min6 cells were exposed to substrate limited media and given 3 mM glucose or 16.7 mM glucose for 45 min followed by 1 μM oligomycin in the high glucose group. Cells were lysed and a fluorescent adduct was generated of malondialdehyde present in cell lysates and thiobarbituric acid that was detected fluorometrically by a Tecan Spark Multi-mode plate reader. Reactions were performed in technical duplicates.

### Luciferin-based ATP determination

Min6 cells were exposed to substrate limited media followed by treatment with low or high glucose as described above. Cells were then lysed using 0.1% tri-chloroacetic acid to terminate ATP consuming reactions. This lysate was neutralized using a Tris–EDTA buffer for use in an ATP luminescence assay (Abcam: 113849). Neutralized cell lysate was introduced into a reaction buffer containing luciferase such that ATP-dependent luminescence could be measured in a microplate format using a Tecan Spark Multi-mode plate reader. Two luciferase reaction samples were measured (technical replicates).

### Oral glucose tolerance testing (OGTT)

OGTT was performed as published^40^; briefly mice were given an oral gavage of glucose totaling 2.5 g/kg and blood glucose was measured by a glucometer at 0-, 15-, 30-, 60-, and 120-min time points. Blood samples were coagulated, serum extracted by centrifugation, and collected for insulin measurements using an Ultra-Sensitive Mouse Insulin ELISA kit (Crystal Chem). For high fat diet mice, additional glucose measurements were taken at 180- and 240-min.

### Intraperitoneal insulin tolerance testing (ITT)

Intraperitoneal injections of insulin were given to mice at 1 unit (insulin)/kg (body weight) and blood glucose was measure at 0-, 15-, 30-, 60-, and 120-min time points. Glucose was measured using a glucometer.

### High fat diet regimen

High fat diet chow (Research diets: D12492) containing 60% kcal from fat was initiated in animals at 4–5 weeks old and continued for 8 weeks with weekly body weight and chow consumption measurements.

### Cardiac function measurements using echocardiography

Echocardiography was performed as previously published^41^ using Vevo2100. Briefly, mice were anesthetized, and M-mode measurements obtained at the level of the papillary muscles in the short-axis. Quantification was performed using VisualSonics software.

### Tissue preparation and immunofluorescence analysis

Organs were fixed in 10% formalin, embedded in paraffin, and sectioned at 5 µm thickness. Antigen retrieval was performed as published^27^. Primary antibody incubation was performed overnight and secondary antibody incubation for 1 h (see Supplementary Table 1 for antibody details). Imaging was performed using a Nikon Digital Sight Ri1 camera equipped on a Nikon Eclipse 50i microscope. Quantification was performed using ImageJ wherein islets were selected as regions of interest and signal calculated as mean grey value minus background mean grey value.

### Statistical analysis

Statistical analysis was performed using GraphPad Prism 9. Unpaired T-tests as well as one-way and two-way ANOVAs were used with Tukey’s method for post-hoc multiple comparisons. P-values of less than 0.05 were considered significant.

### Ethical approval statement

All animal use in this study was reviewed and approved by the Institutional Animal Care and Use Committee (IACUC) at Drexel University College of Medicine, Philadelphia, PA. All authors approved the final version of the manuscript submitted. None of the authors have any professional or financial affiliations that may be perceived to have biased presentation.

## Supplementary Information

Supplementary Information.

## Abbreviations

GPCR: G-protein coupled receptor
GRK2: GPCR kinase 2
GLUT-2: Glucose transporter 2
IBMX: 3-Isobutyl-1-methylxanthine
FCCP: Carbonyl cyanide-4-phenylhydrazone
R/A: Rotenone/antimycin A
HFD: High fat diet
PTx: Pertussis toxin
OCR: Oxygen consumption rate
α2AR: Alpha2 adrenergic receptor
pancGRK2KO: Pancreatic-specific GRK2 knockdown
VAT: Visceral adipose tissue
SAT: Subcutaneous adipose tissue
BAT: Brown adipose tissue
mPTP: Mitochondrial permeability transition pore
HF: Heart failure
Utx: Untreated
siCtrl: Scrambled oligonucleotides
siGRK2: Silencing oligonucleotides for GRK2
BCA: Bicinchoninic acid
ATP: Adenosine triphosphate
KD: Knockdown
KO: Knockout
IF: Immunofluorescence
