# Supplementary material for: GRK2 contributes to glucose mediated calcium responses and insulin secretion in pancreatic islet cells
Source: Sci Rep. 2021 May 27;11:11129. doi: 10.1038/s41598-021-90253-z (PMC8159944; doi:10.1038/s41598-021-90253-z)
Supplement: Supplementary file 1 — Supplementary Information. [file 41598_2021_90253_MOESM1_ESM.pdf]

GRK2 contributes to glucose mediated calcium responses and insulin secretion in pancreatic islet cells.

Jonathan Snyder[1], Atreju I. Lackey[1], G. Schuyler Brown[1], Melisa Diaz[1], Tian Yuzhen[1], Priscila Y. Sato[1]*

1. Drexel University College of Medicine, Department of Pharmacology and Physiology, Philadelphia, PA 19102

* Corresponding Author:

Priscila Y Sato
Drexel University College of Medicine
Department of Pharmacology and Physiology
245 N 15th Street
NCB 8119
Philadelphia, PA
19102
Phone: 267.359.2667
Fax: 215.762.2299
Pys26@drexel.edu

Supplemental Figure Legends:

Supplementary Fig. 1: (**A-B**) Full-length representative Western blot for GRK2 with β-actin loading control in Min6 cells. (**C**) Quantitative real time PCR results for other GRK isoforms in Min6 cells untreated or treated with scrambled silencing oligonucleotide (siCtrl) or GRK2 silencing oligonucleotide (siGRK2) normalized to UTx GRK2 expression (n=10 preparations/group in 5 independent cultures, 18s was used as housekeeping gene. (**D**) Quantification of peak Fluo-8 signal following IBMX treatment during calcium imaging experiment. (n=48-60 cells/group in 4 independent experiments). (**E**) Representative trace of calcium imaging experiment in the absence of extracellular calcium in Min6 cells. (**F**) Glucose-stimulated insulin secretion in cells treated with IBMX (n=12/group in 4 independent experiments). (**G**) cAMP measurements in Min6 cells exposed to high glucose in the presence of IBMX (n=3/group in 3 independent experiments). All data shown are mean +/-SEM. *p≤0.05; ***p≤0.001.

Supplementary Fig. 2: (**A**) Quantitative real time PCR results for UCP3, mature insulin, and premature insulin in Min 6 cells untreated (Utx) or treated with a scrambled oligonucleotide (siCtrl) or a GRK2-silencing oligonucleotide (siGRK2) (n=8 preparations/group in 8 independent cell cultures), 18s was used as housekeeping gene. (**B**) Reactive oxygen species (ROS) measurements in Utx, siCtrl, siGRK2 cells exposed to low or high glucose levels (n=7 preparations/group in 7 independent cell cultures). (**C**) Lipid peroxidation measurements in Utx, siCtrl, and siGRK2 cells (n=5-6 preparations/group in 5-6 independent cell cultures). (**D**) Expression of electron transport chain proteins (n=7 preparations/group in 7 independent cell cultures) (**E-F**) Representative Western blot for (D), TOM20 was used as loading control. (**G**) Expression of ATP synthase subunit c (ATP5C) (n=3-4 samples/group in 4 independent cell cultures) (**H-I**) Representative Western blot for ATP5C normalized to TOM20. All data shown are mean+/-SEM. *p≤0.05; **p≤0.01; ***p≤0.001.

Supplementary Fig. 3: Full-length Western blot image for GRK2 and β-actin corresponding to Fig 3A.

Supplementary Fig. 4: (**A**) Quantification of GRK2 Western blots in isolated islets from control or pancGRK2KO mice (n=9 animals/group). (**B**) Overall tracing of blood glucose levels during oral glucose tolerance test (OGTT) in Cre (-) flox/flox (solid circles) or Cre (+) WT/WT (solid squares), two of the possible control genotypes (n=10-12 animals/group). (**C**) Overall glucose recordings during an insulin tolerance test

(ITT) in control (solid circles) or pancGRK2KO animals (solid squares) (n=6-11 animals/group). (**D**) Quantification of glucagon signal (n=25-37 islets/group in 4-5 animals/group). (**E**) Representative glucagon immunofluorescence staining. (**F**) Quantification of percent islet area stained positive for glucagon (same n as D). (**G**) Quantitative real time PCR results from isolated islets for mature insulin, premature insulin, and alpha2a-receptor (n=5-7 sample/group in 10-14 animals/genotype). (**H**) Heart weight (HW) to tibia length (TL) ratios in control (black) and pancGRK2KO (grey) animals (n=14-22 mice/group). All data shown are mean+/-SEM. ****p≤0.0001.

Supplementary Fig. 5: (**A**) Food consumption per week from animals exposed to HFD regimen (n=24-48 measurements/group from 3 independent cohorts). (**B**) Overall glucose recordings during an insulin tolerance test (ITT) in control HFD (solid circles) or pancGRK2KO HFD animals (solid squares; n=3-4 animals/group) (**C**) Islet glucagon immunofluorescence signal quantification in control and pancGRK2KO animals subjected to HFD regimen (n=26-31 islets/group in 4-5 animals/group). (**D**) Percent area of islet stained positive for glucagon expression in control and pancGRK2KO animals subjected to HFD (same n values as (C)). (**E**) Representative images of glucagon immunofluorescence staining in control and pancGRK2KO mice subjected to HFD. representative images for glucagon immunofluorescence. All data shown are mean+/-SEM. **p≤0.01.

Supplementary Table 1: Antibodies used for Western blots and/or immunofluorescence experiments.

Supplementary Table 2: Primer sequences used in qRT-PCR experiments.

Supplementary Table 3: Assay IDs for Taqman Gene expression experiments.

Supplementary Fig. 1

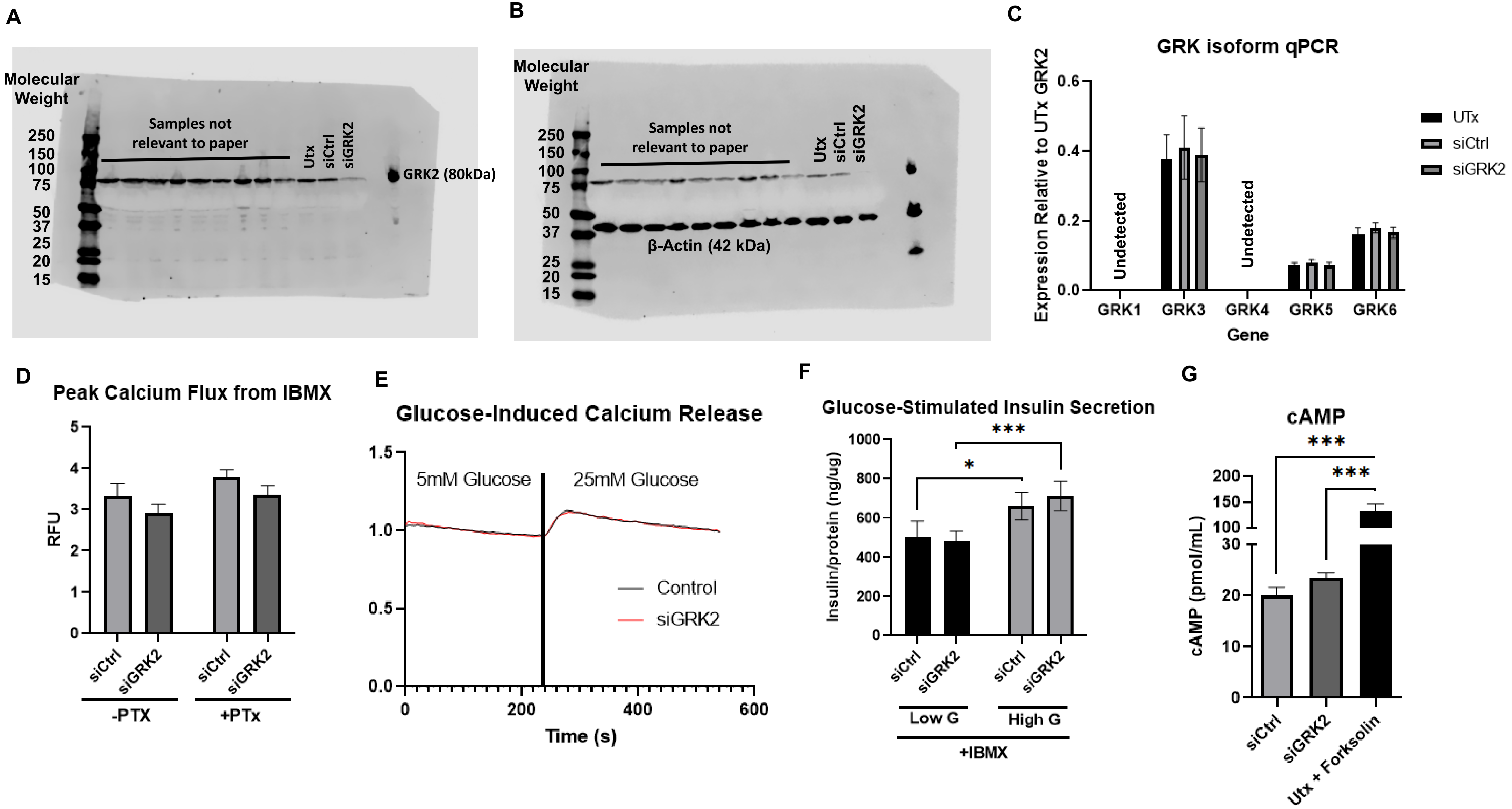

Supplementary Fig. 2

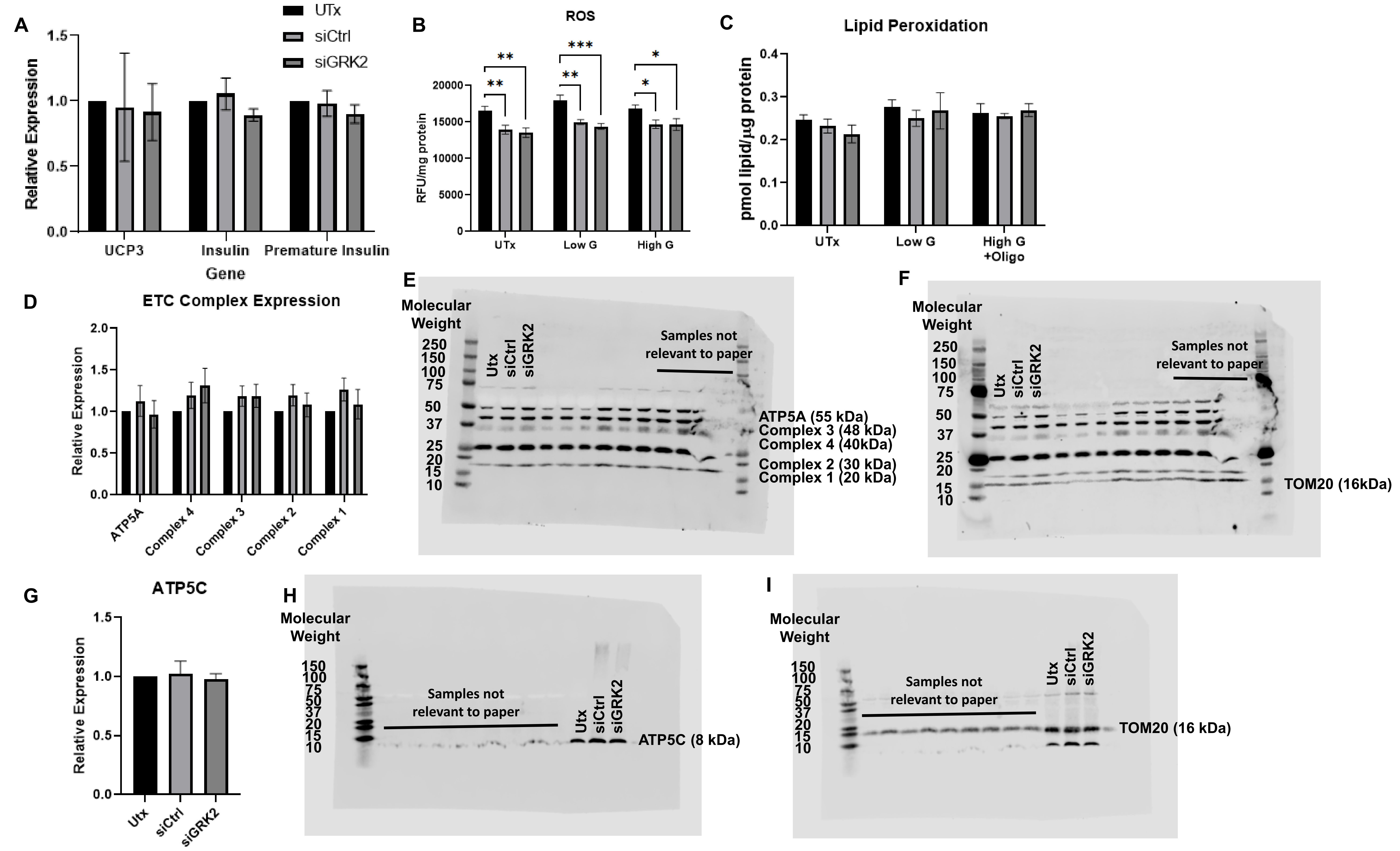

Supplementary Fig. 3

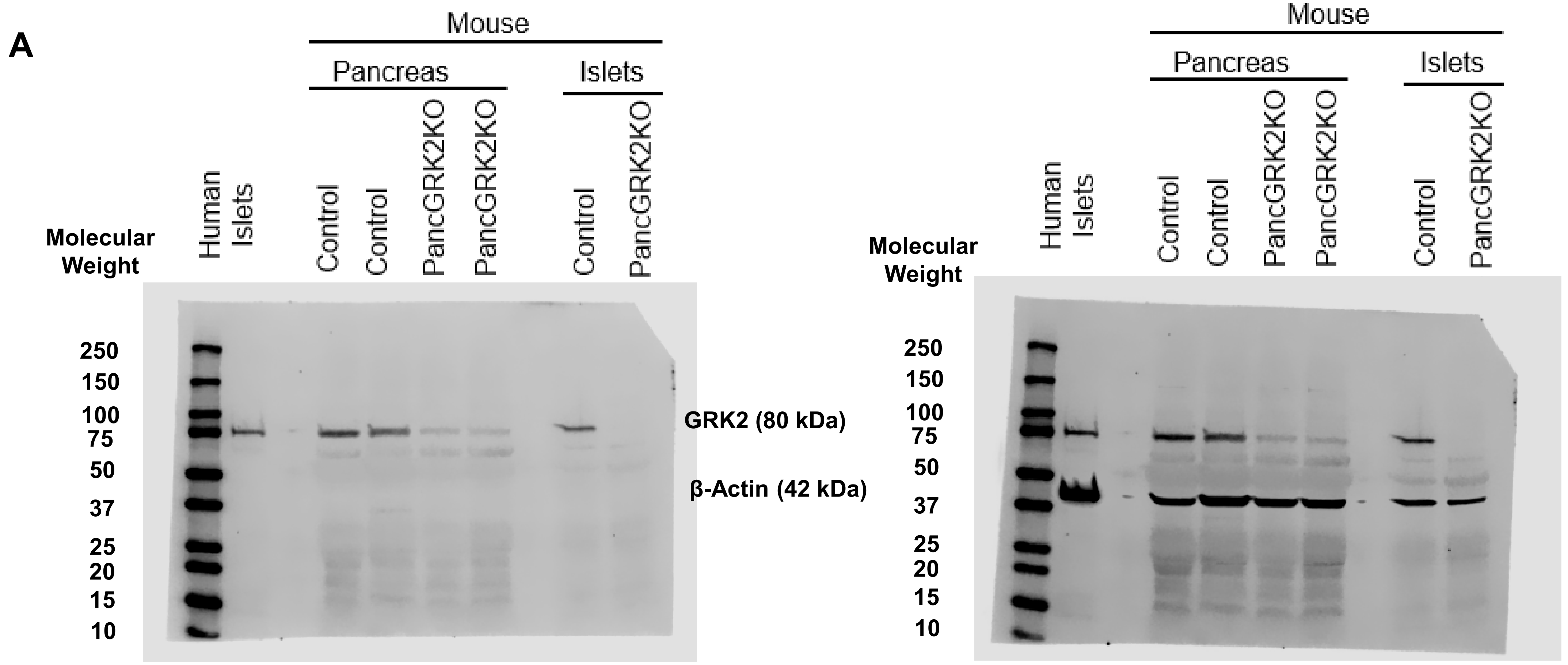

Supplementary Fig. 4

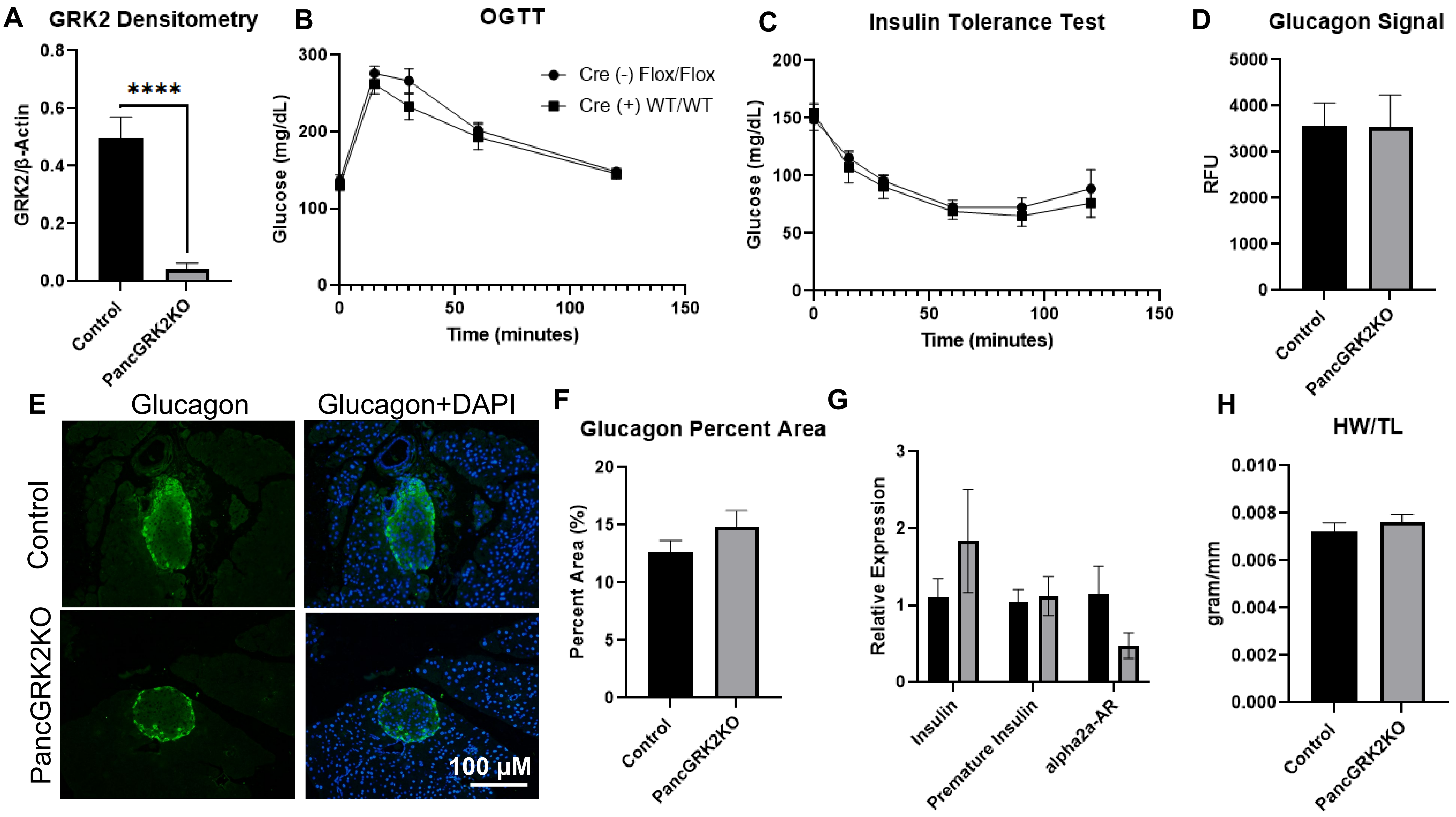

Supplementary Fig. 5

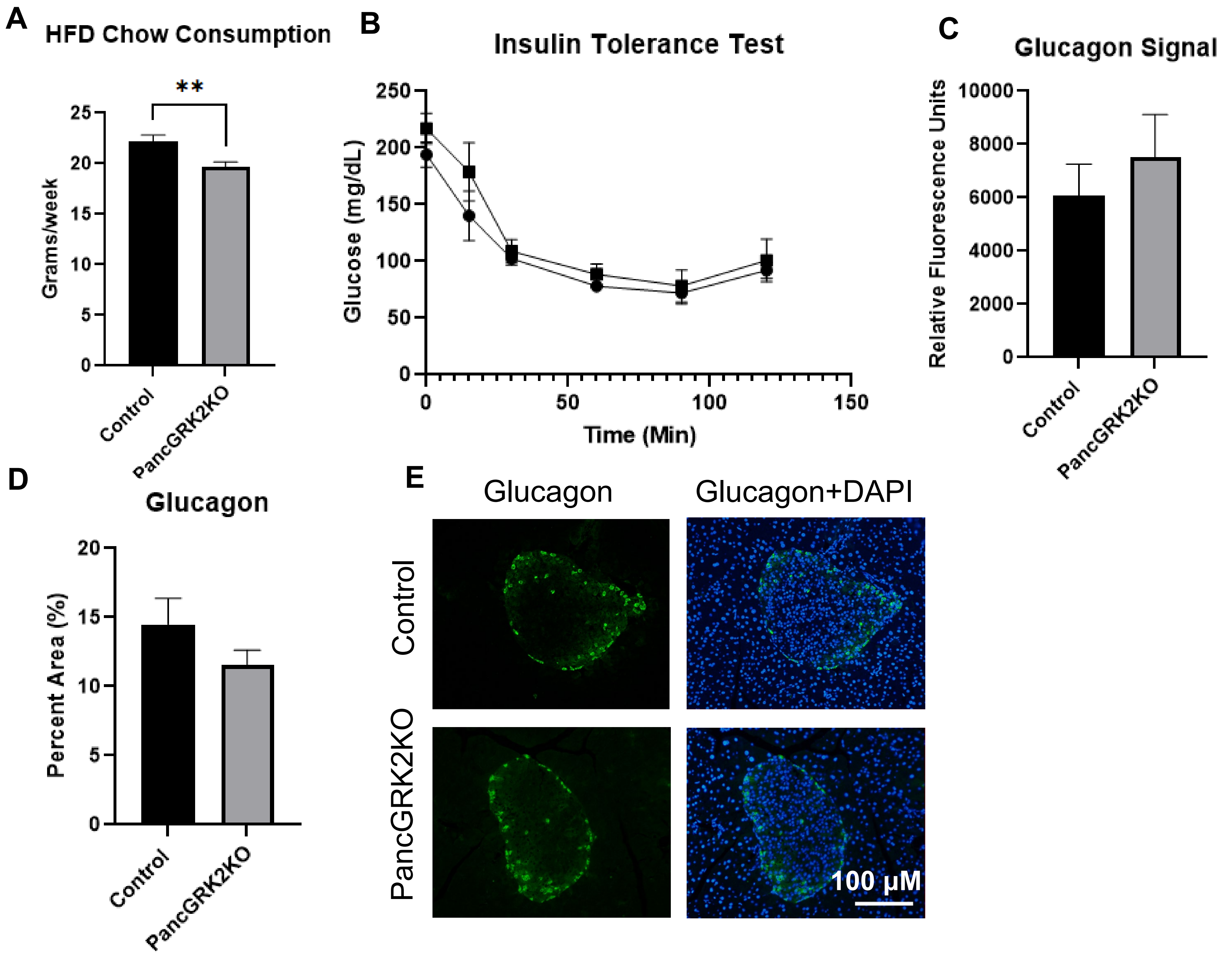

Supplementary Table 1

Antibody Target	Dilution Factor	Vendor (Cat#)
GRK2	5,000	Sigma (G0296)
B-Actin	5,000	Santa-Cruz (sc-47778)
OxPhos Cocktail	250	Abcam (ab110413)
ATP5C	5,000	Abcam (ab181243)
TOM20	5,000	Proteintech (11802-1-AP)
Insulin	50	Abcam(ab7842)
Glucagon	2000	Abcam (ab92517)
Anti-Guinea Pig-Cy3	500	Jackson Immuno Research (706-165-148)
Anti-Rabbit Alexafluor488	500	Abcam(ab150077)
Anti-Rabbit Alexafluor680	10,000	LI-COR (925-68071)
Anti-Rabbit Alexafluor800	10,000	LI-COR (925-32211)
Anti-Mouse Alexafluor680	10,000	Invitrogen (A21058)
Anti-Mouse Alexafluor800	10,000	LI-COR (925-32210)

Supplementary Table 2

Primer	Sequence (5’-3’)
18S-For	GGCCGGTTCTTAGTTGGTGGAGCG
18S-Rev	CTGAACGCCACTTGTCCCTC
Premature Insulin-For	TGGCTTCTTCTACACACCCAAG
Premature Insulin-Rev	ACAATGCCACGCTTCTGCC
Insulin-For	TGGCTTCTTCTACACACCCAAG
Insulin-Rev	ACAATGCCACGCTTCTGCC
UCP2-For	TCCCCTGTTGATGTGGTCAA
UCP2-Rev	CAGTGACCTGCGCTGTGGTA
UCP3-For	CCTACGACATCATCAAGGAGAAGTT
UCP3-Rev	TCCAAAGGCAGAGACAAAGTGA
Alpha2a-AR-For	TGGACCAAGACA GAAGGAAATGA
Alpha2a-AR-Rev	CAAGTGGTGCCTCAGCGAAT

Supplementary Table 3

Gene	Thermo-fisher Taqman Assay ID
GRK1	Mm01220712_m1
GRK2	Mm00804778_m1
GRK3	Mm00622042_m1
GRK4	Mm01213690_m1
GRK5	Mm00517039_m1
GRK6	Mm00442425_m1
18S	Mm02619580_g1
